# Revisiting a natural wine salt: calcium (2*R*,3*R*)-tar­trate tetra­hydrate

**DOI:** 10.1107/S2053229624008015

**Published:** 2024-09-04

**Authors:** Alvaro Polo, Alejandro Soriano-Jarabo, Ricardo Rodríguez, Ramón Macías, Pilar García-Orduña, Pablo J. Sanz Miguel

**Affiliations:** a Departamento de Química Inorgánica, Instituto de Síntesis Química y Catálisis Homogénea (ISQCH), Universidad de Zaragoza–CSIC, 50009 Zaragoza, Spain; Universidade Federal de Minas Gerais, Brazil

**Keywords:** crystal structure, wine crystal, calcium tar­trate, hy­dro­gen bonding, chirality, hydration

## Abstract

The crystal structure of the salt calcium (2*R*,3*R*)-tar­trate tetra­hydrate {sys­tem­atic name: poly[[di­aqua­[μ_4_-(2*R*,3*R*)-2,3-di­hydroxy­butane­dioato]calcium(II)] di­hydrate]}, {[Ca(C_4_H_8_O_8_)(H_2_O)_2_]·2H_2_O}_*n*_, is reported. The absolute configuration of the crystal was established unambiguously using anomalous dispersion effects in the diffraction patterns. High-quality data also allowed the location and free refinement of all the H atoms, and therefore to a careful analysis of the hy­dro­gen-bond inter­actions.

## Abstract 2

In the salt calcium (2*R*,3*R*)-tar­trate tetra­hydrate, the absolute configuration was established unambiguously using anomalous dispersion effects in the diffraction patterns. High-quality data also allowed the location and free refinement of all the H atoms.

## Introduction

The opening of a bottle of wine is a process that can elicit a variety of expectations, either in terms of the wine’s taste, colour, smell, sensations or even in the occasional discovery of brilliant crystals, typically found on the surface of the cork in contact with the wine. The so-called *Weinsteine* or *wine diamonds* (Derewenda, 2008 ▸) are regarded by winemakers as a sign of quality, as their presence indicates that wine has been handled with natural methods and proper timing. It is known that such *diamonds* are actually crystalline tar­trate salts.

**Figure 1:**
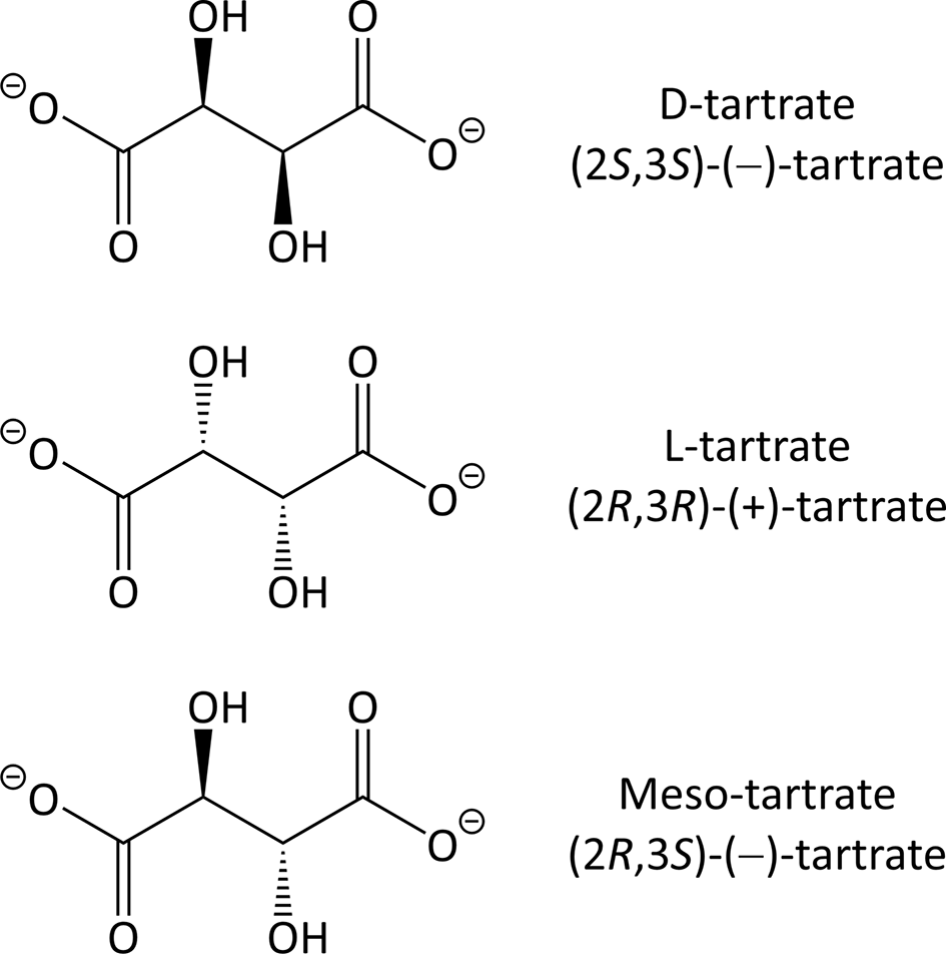
The enanti­omeric and meso forms of tar­trate.

Tartaric acid (Astbury, 1923 ▸), also known as 2,3-di­hy­droxy­butane­dioic acid, is a naturally occurring substance that is typically found on grapes and other plants. Although two enanti­omers (2*R*,3*R*/2*S*,3*S*) and a meso form (2*S*,3*R*/2*R*,3*S*) are possible, only the 2*R*,3*R* enanti­omer, namely l-(+)-tartaric acid, is biologically produced by vining plants. Deprotonation to its tar­trate form (Fig. 1 ▸) during the fermentation and aging steps of wine production in the presence of alkali earth metal cations, usually K^+^ and Ca^2+^, may result in the slow crystallization of 2*R*,3*R* salts. This process can extend over a pro­longed period, frequently becoming noticeable after com­mercial release.

Pioneering studies on the unit-cell parameters of the title com­pound, Ca[(2*R*,3*R*)-C_4_H_4_O_6_]·4H_2_O (**1**), were reported by Evans (1935 ▸), yielding a *P*2_1_2_1_2_1_ space group crystal structure, with unit-cell parameters *a* = 9.20 (2), *b* = 10.54 (2) and *c* = 9.62 (2) Å. Several studies since then have confirmed the crystal structure of this salt, corroborating the space group and unit-cell dimensions (Ambady, 1968 ▸; Hawthorne *et al.*, 1982 ▸; Boese & Heinemann, 1993 ▸; Kaduk, 2007 ▸). In all the studies, aqueous solutions of tartaric acid were employed, from which crystals were grown. Although tartaric acid and its derivatives, especially its sodium ammonium salt, have long been central to the analysis of stereochemistry and chirality (Gal, 2008 ▸), since the pioneering works of Pasteur and Biott (see Flack, 2009 ▸, and references therein), it is important to note that in none of these structural reports about Ca(C_4_H_4_O_6_)·4H_2_O was it possible to identify which of the enanti­omers was being measured through anomalous dispersion effects.

Inter­estingly, triclinic polymorphs of racemic **1** (*i.e.* with both enanti­omers in the unit cell) have also been reported (Le Bail *et al.*, 2009 ▸; Appelhans *et al.*, 2009 ▸; Fukami *et al.*, 2016 ▸). Furthermore, not only polymorphs, but also hydrates and solvates of Ca and tar­trate have been reported. In this context, calcium tar­trate has been found to also crystallize as its anhydrous (Appelhans *et al.*, 2009 ▸; Aljafree *et al.*, 2024 ▸), trihydrate (de Vries & Kroon, 1984 ▸) and hexa­hydrate forms (Ventruti *et al.*, 2015 ▸), and has been observed to cocrystallize with other species (Wartchow, 1996 ▸). The absolute configuration of these hydrates and solvates has been established experimentally, except in the case of the trihydrate form, which was found to contain the *meso*-tartaric form. Obviously, different hydration is related to dissimilar connectivity and crystal packing.

Here, we report the crystal structure of the calcium (2*R*,3*R*)-tar­trate tetra­hydrate salt (**1**), obtained from a crystal which was found and picked up from the cork of a *Crianza* red wine bottle from D.O. Campo de Borja (2016). This tetra­hydrated salt crystallizes in the ortho­rhom­bic space group *P*2_1_2_1_2_1_, with the unit-cell dimensions [*a* = 9.1587 (4), *b* = 9.5551 (4) and *c* = 10.5041 (5) Å], which are close to those reported by Evans (1935 ▸). High-quality experimental diffraction data allowed us to establish unambiguously the absolute structure and therefore the absolute configuration of the salt, and to analyze inter­molecular inter­actions in the crystal packing.

## Experimental

### Single-crystal selection

Single crystals were found in the cork of a wine bottle, removed and selected under a microscope.

### Single-crystal X-ray diffraction

Crystal data, data collection and structure refinement details are summarized in Table 1 ▸. H atoms were located in dif­ference Fourier maps and freely refined. High-quality and com­plete diffraction data, with 99.2% of the reflections measured until a maximal resolution of (sin θ/λ)_max_ = 0.667 Å^−1^ (with almost all the Friedel pairs: number of Friedel pairs measured out to the maximal resolution divided by the num­ber of theoretically possible is 0.981, very close to unity), a mean redundancy higher than 20 and a good agreement factor (*R*_int_ = 0.030) of this Ca-containing crystal allowed us to establish the absolute structure in the solid state and therefore the absolute configuration of the mol­ecule. For that purpose, the Flack parameter (Flack & Bernardinelli, 1999 ▸, 2000 ▸) has been refined. The obtained values are 0.028 (19) by classical fit to all intensities and 0.023 (3) using 937 quotients (Parsons *et al.*, 2013 ▸). The ob­tained values of the parameter and its standard uncertainty (s.u.) value provide evidence for a strong inversion-distinguishing power and a correct estimation of the absolute structure for this structural model.

## Results and discussion

The asymmetric unit of Ca[(2*R*,3*R*)-C_4_H_4_O_6_]·4H_2_O (**1**) is formed by a Ca^2+^ ion, a tar­trate ligand and four water mol­ecules. In the crystal structure, the tar­trate ion exhibits typical bonding connections (Ambady, 1968 ▸). Salient bond distances and angles are listed in Tables S1 and S2 of the supporting information. The two C—O bonds of each carboxyl­ate group, which, along with the hy­droxy substituents, chelate two Ca^2+^ cations, are significantly longer than the other two carboxyl­ate C—O bonds, where the O atoms bind to additional adjacent Ca atoms [C1—O11 = 1.2659 (14) Å and C4—O41 = 1.2681 (14) Å *versus* C1—O12 = 1.2483 (15) Å and C4—O42 = 1.2472 (14) Å]. All the C atoms of the tar­trate skeleton exhibit similar C—C separations, and are positioned in an almost coplanar manner, with maximal deviations from the best plane of 0.0020 (6) Å. It is noteworthy that the folding of this di­carboxyl­ate entity is asymmetrical. Specifically, the O21 atom of the alcohol group lies nearly in the plane defined by the C1 atom and the atoms coordinated to its *sp*^2^ hybridization, namely, C1, C2, O11 and O12 [0.069 (2) Å], whereas the alcohol O31 atom is placed significantly out of the analogous plane [atoms C3, C4, O41 and O42, 0.575 (2) Å].

### Ca environment

**Figure 2:**
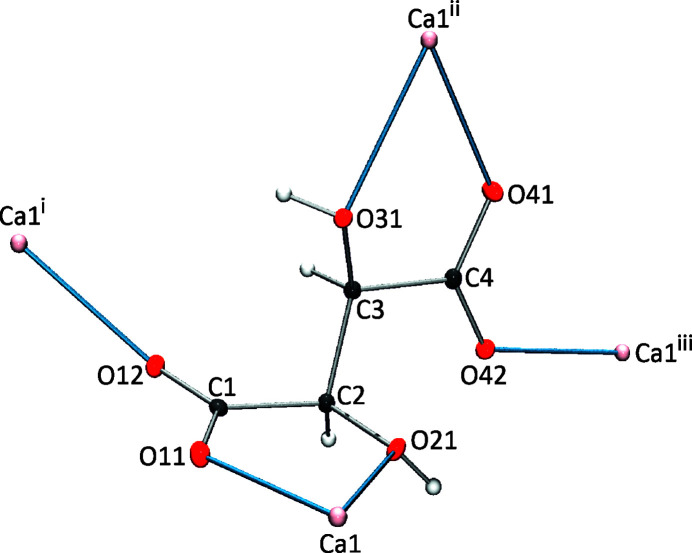
Ca^2+^ cations bonded to a tar­trate anion in **1**. [Symmetry codes: (i) −*x*, *y* + 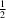, −*z* + 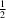; (ii) −*x* + 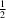, −*y* + 1, *z* + 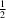; (iii) −*x* + 1, *y* + 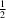, −*z* + 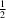.]

**Figure 3:**
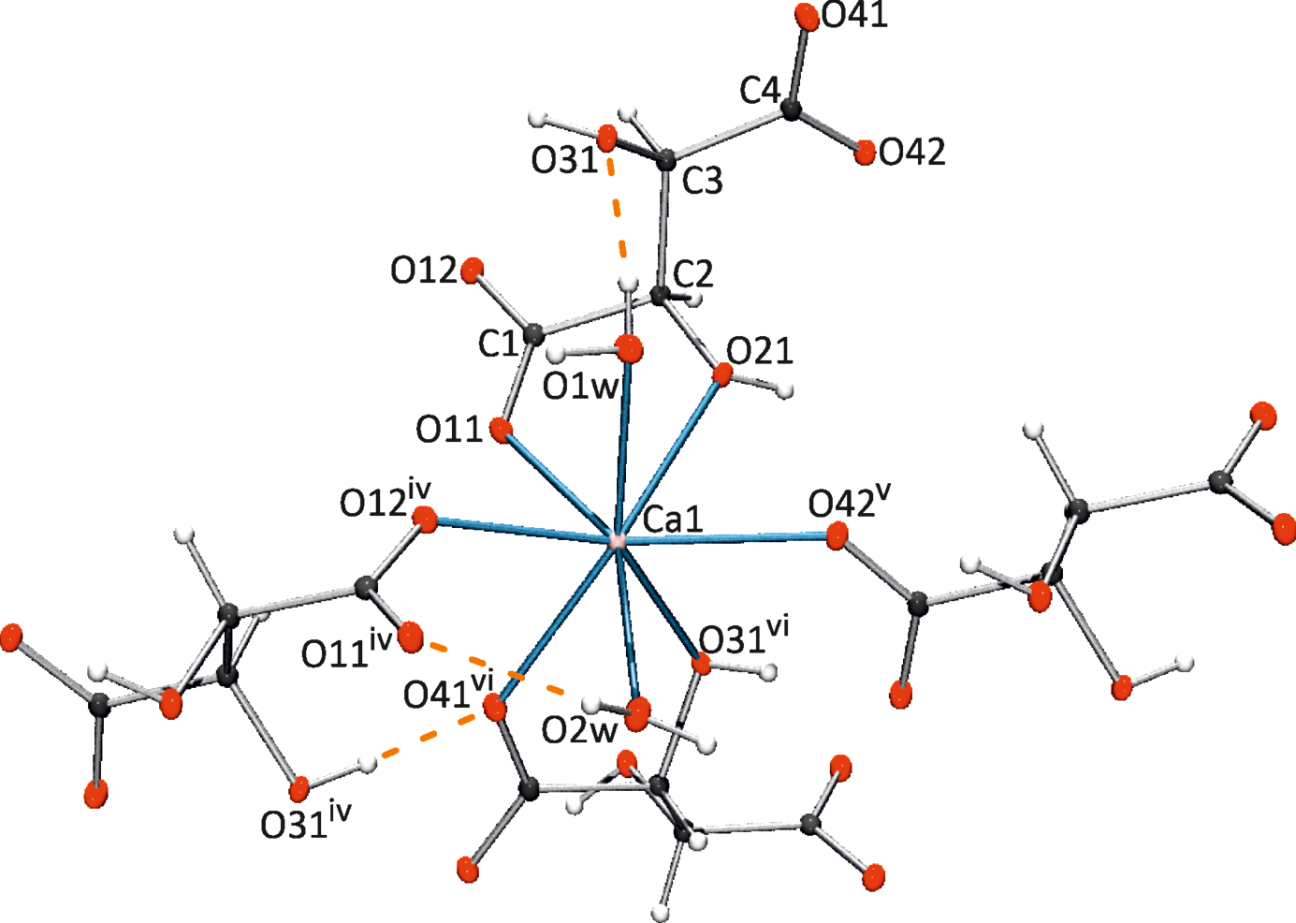
Coordination sphere of the Ca^2+^ cation in **1**. [Symmetry codes: (iv) −*x*, *y* − 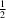, −*z* + 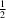; (v) −*x* + 1, *y* − 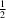, −*z* + 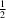; (vi) −*x* + 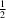, −*y* + 1, *z* − 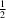.

In the crystal packing, each tar­trate anion acts as a tetra­topic ligand, serving as a chelate for two Ca^2+^ cations and as a terminal ligand for two additional Ca^2+^ cations (Fig. 2 ▸), whereas the Ca^2+^ cations (Ca1) are coordinated to four sym­metry-related tar­trate anions and two water mol­ecules in a distorted pseudo-octa­hedral coordination environment (Fig. 3 ▸).

Among the eight coordination sites of Ca, two are occupied by monodentate O atoms from carb­oxy­late groups [O12—Ca1—O42 = 137.72 (3)°], with another two sites hosting water mol­ecules [O1*W*—Ca1—O2*W* = 97.34 (3)°]. The coordination sphere of Ca1 is com­pleted by two chelating tar­trate ligands bonded by different edges, namely, O11—C1—C2—O21 and O31—C3—C4—O41. In both chelates, separation from the deprotonated O atoms to the Ca^2+^ cation [Ca1—O11 = 2.3733 (8) Å and Ca1—O41 = 2.4137 (9) Å] are significantly shorter com­pared to those of the alcohol groups [Ca1—O21 = 2.4544 (9) Å and Ca1—O31 = 2.5102 (9) Å]. These Ca—O distances range from 2.3733 (8) (Ca1—O11) to 2.5102 (9) Å (Ca—O31), which are consistent with the expected values (Ambady, 1968 ▸). It is noteworthy that this coordination of the Ca^2+^ ion in **1** notably differs from that of the triclinic polymorph, where the eight-coordinated Ca^2+^ ion is bound to two bis-chelated tar­trate ligands and four water mol­ecules.

### Hydrogen bonding

**Figure 4:**
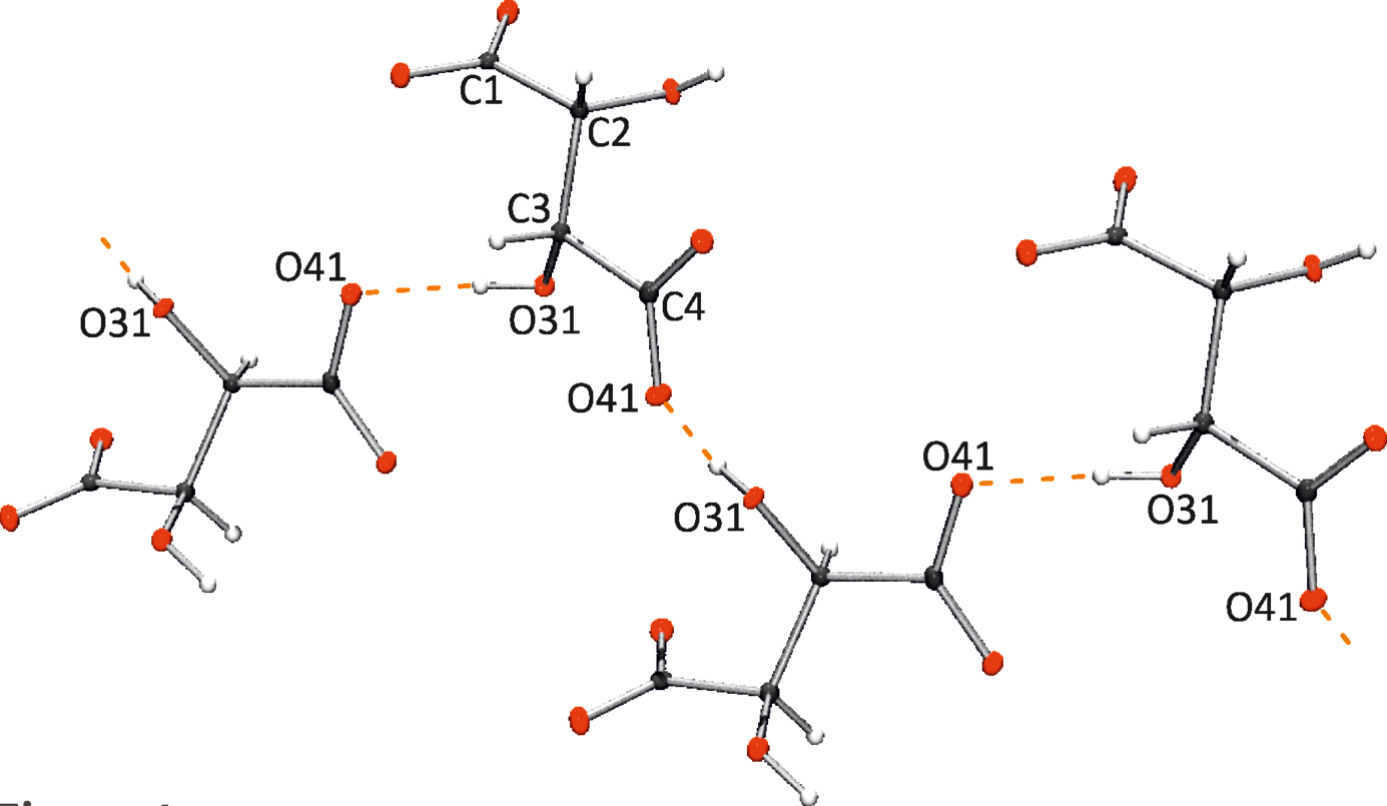
Hydrogen bonding involving tar­trate anions in **1**. Calcium cations and water mol­ecules have been omitted for clarity.

The two additional water mol­ecules fulfilling the unit cell of **1**, and which are not coordinated to Ca, are involved in hy­dro­gen-bonding inter­actions. The crystal lattice is mainly stabilized by electrostatics and hy­dro­gen bonding. The tar­trate anions are connected *via* short hy­dro­gen bonds [O31—H31⋯O41 = 2.5529 (12) Å] in a zigzag fashion along the *a* axis (Fig. 4 ▸). Finally, water mol­ecules participate in eight additional hy­dro­gen bonds involving tar­trate anions and other water mol­ecules (Table 2 ▸).

## Summary

The title calcium (2*R*,3*R*)-tar­trate tetra­hydrate salt (**1**) crystallized in the ortho­rhom­bic space group *P*2_1_2_1_2_1_, as anti­cipated by Evans (1935 ▸). In this work, anomalous dispersion effects in the crystal diffraction patterns led to the determination of the absolute configuration of the l-(+)-tar­trate salt **1**. The absolute configuration has been resolved on the basis of anomalous dispersion effects in the crystal diffraction patterns and matches the enanti­omer expected from a natural wine-making process. The good crystal quality allowed for precise determination of the geometrical arrangement, particularly enabling the localization of H atoms, and therefore the observation and accurate characterization of the hy­dro­gen-bonding network.

## Supplementary Material

Crystal structure: contains datablock(s) I, global. DOI: 10.1107/S2053229624008015/dg3060sup1.cif

Structure factors: contains datablock(s) I. DOI: 10.1107/S2053229624008015/dg3060Isup2.hkl

Additional tables. DOI: 10.1107/S2053229624008015/dg3060sup3.pdf

CCDC reference: 2377585

## Figures and Tables

**Table 1:** Experimental details

Crystal data	
Chemical formula	[Ca(C_4_H_4_O_6_)(H_2_O)_2_]·2H_2_O
*M*_r_	260.22
Crystal system, space group	Orthorhombic, *P*2_1_2_1_2_1_
Temperature (K)	100
*a*, *b*, *c* (Å)	9.1587 (4), 9.5551 (4), 10.5041 (5)
*V* (Å^3^)	919.24 (7)
*Z*	4
Radiation type	Mo *K*α
μ (mm^−1^)	0.73
Crystal size (mm)	0.15 × 0.13 × 0.09

Data collection	
Diffractometer	Bruker D8 VENTURE
Absorption correction	Multi-scan (*SADABS*; Bruker, 2016 ▸)
*T*_min_, *T*_max_	0.889, 0.937
No. of measured, independent and observed [*I* > 2σ(*I*)] reflections	47365, 2275, 2268
*R*_int_	0.030
(sin θ/λ)_max_ (Å^−1^)	0.667

Refinement	
*R*[*F*^2^ > 2σ(*F*^2^)], *wR*(*F*^2^), *S*	0.014, 0.035, 1.09
No. of reflections	2275
No. of parameters	184
H-atom treatment	All H-atom parameters refined
Δρ_max_, Δρ_min_ (e Å^−3^)	0.28, −0.25
Absolute structure	Flack *x* determined using 937 quotients [(*I*^+^) − (*I*^−^)]/[(*I*^+^) + (*I*^−^)] (Parsons *et al.*, 2013 ▸)
Absolute structure parameter	0.023 (3)

Computer programs: *SAINT* in *APEX3* (Bruker, 2016 ▸), *SHELXS2013* (Sheldrick, 2008 ▸), *SHELXL2018* (Sheldrick, 2015 ▸), *ORTEP-3 for Windows* (Farrugia, 2012 ▸) and *XCIF* in *PLATON* (Spek, 2020 ▸).

**Table 2:** Hydrogen-bond geometry (Å, °)

*D*—H⋯*A*	*D*—H	H⋯*A*	*D*⋯*A*	*D*—H⋯*A*
O31—H31⋯O41^vii^	0.84 (3)	1.71 (3)	2.5529 (12)	174 (2)
O21—H21⋯O4*W*	0.83 (2)	1.88 (2)	2.7023 (13)	174 (2)
O1*W*—H2*W*⋯O31	0.82 (3)	2.11 (3)	2.9236 (13)	170 (2)
O2*W*—H3*W*⋯O3*W*	0.82 (2)	1.95 (2)	2.7483 (13)	164 (2)
O2*W*—H4*W*⋯O11^viii^	0.87 (2)	2.12 (2)	2.8658 (13)	144 (2)
O3*W*—H5*W*⋯O42^ix^	0.90 (3)	2.26 (3)	3.0318 (13)	144 (2)
O3*W*—H6*W*⋯O11^x^	0.80 (2)	2.09 (2)	2.8809 (13)	168 (2)
O4*W*—H7*W*⋯O2*W*^x^	0.77 (3)	2.16 (3)	2.9199 (14)	170 (2)
O4*W*—H8*W*⋯O1*W*^xi^	0.83 (3)	2.31 (3)	3.1263 (15)	171 (2)

Symmetry codes: (vii) 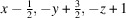; (viii) 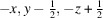; (ix) 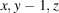; (x) 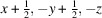; (xi) 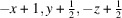.
