# Supplementary material for: Revisiting a natural wine salt: calcium (2R,3R)-tar­trate tetra­hydrate
Source: Acta Crystallogr C Struct Chem. 2024 Sep 4;80(Pt 10):681–4. doi: 10.1107/S2053229624008015 (PMC11451013; doi:10.1107/S2053229624008015)
Supplement: Supplementary file 3 [file c-80-00681-sup3.pdf]

Acta Cryst C STRUCTURAL CHEMISTRY

Volume 80 (2024)

Supporting information for article:

Revisiting a natural wine salt: calcium (2*R*,3*R*)-tartrate tetrahydrate

Alvaro Polo, Alejandro Soriano, Ricardo Rodríguez, Ramón Macías, Pilar García-Orduña and Pablo J. Sanz Miguel

Table S1 Bond lengths [Å] in Ca[2*R*,3*R*-$C_4H_4O_6$]·$4H_2O$ (**1**).

Ca1-O11	2.3733(8)	C1–C2	1.5330(15)
Ca1-O12	2.4015(9)	C1–O11	1.2659(14)
Ca1–O21	2.4544(9)	C1–O12	1.2483(15)
Ca1–O31	2.5102(9)	C2–C3	1.5220(15)
Ca1–O41	2.4137(9)	C2–O21	1.4229(13)
Ca1–O42	2.4784(9)	C3–C4	1.5313(16)
Ca1–O1w	2.4900(9)	C3–O31	1.4312(13)
Ca1-O2w	2.4371(9)	C4–O41	1.2681(14)
		C4–O42	1.2472(14)

Table S2 Bond angles [°] in Ca[2*R*,3*R*-$C_4H_4O_6$]·$4H_2O$ (**1**).

O11–Ca1–O12	77.52(3)	O31–Ca1–O42	78.48(3)
O11–Ca1–O21	65.82(3)	O31–Ca1–O1w	149.66(3)
O11–Ca1–O31	89.30(3)	O31–Ca1–O2w	96.36(3)
O11–Ca1–O41	79.05(3)	O41–Ca1–O42	131.06(3)
O11–Ca1–O42	132.76(3)	O41–Ca1–O1w	145.89(3)
O11–Ca1–O1w	92.21(3)	O41–Ca1–O2w	77.10(3)
O11–Ca1–O2w	149.92(3)	O42–Ca1–O1w	78.36(3)
O12–Ca1–O21	126.25(3)	O42–Ca1–O2w	77.23(3)
O12–Ca1–O31	138.76(3)	O11–C1–O12	125.61(10)
O12–Ca1–O41	75.20(3)	O12–C1–C2	116.20(10)
O12–Ca1–O42	137.72(3)	O11–C1–C2	118.18(10)
O12–Ca1–O1w	70.71(3)	O21–C2–C3	111.42(9)
O12–Ca1–O2w	78.88(3)	O21–C2–C1	109.84(9)
O1w–Ca1–O2w	97.34(3)	C3–C2–C1	109.08(9)
O21–Ca1–O31	80.26(3)	O31–C3–C2	111.45(9)
O21–Ca1–O41	129.72(3)	O31–C3–C4	108.95(9)
O21–Ca1–O42	67.18(3)	C2–C3–C4	113.68(9)
O21–Ca1–O1w	72.84(3)	O42–C4–O41	125.35(11)
O21–Ca1–O2w	144.25(3)	O42–C4–C3	119.59(10)
O31–Ca1–O41	63.91(3)	O41–C4–C3	114.95(10)
